# Hematologic health services and practical characteristics: report of a nationwide survey among Chinese hematologists

**DOI:** 10.1186/s12913-024-10829-z

**Published:** 2024-03-12

**Authors:** Jia Chen, Jiali Gu, Yuhua Ru, Jianxiang Wang, Yu Hu, Kaiyan Liu, Qifa Liu, Xiaohui Zhang, Zhijian Xiao, Weili Zhao, Yang Xu, Xiaojun Huang, Depei Wu, on behalf of Chinese Society of Hematology, Chinese Society of Hematologist and Chinese Society of Hematology Youth Committee

**Affiliations:** 1 National Clinical Research Center for Hematologic Diseases, Jiangsu Institute of Hematology, The First Affiliated Hospital of Soochow University, 188 Shizi Street, Suzhou, Jiangsu Province 215006 China; 2 Institute of Hematology and Blood Diseases Hospital, Chinese Academy of Medical Sciences & Peking Union Medical College, 288 Nanjing Road, Tianjin, China; 3 Institute of Hematology, Union Hospital, Tongji Medical College, Huazhong University of Science and Technology, 1095 Jiefang Avenue, Wuhan, China; 4 Peking University Institute of Hematology, Peking University People’s Hospital, Peking University, 11 Xizhimen South Street, Xicheng District, Beijing, 100044 China; 5 Department of Hematology, Nanfang Hospital, Southern Medical University, 1838 Guangzhou Avenue, Guangzhou, China; 6 State Key Laboratory of Medical Genomics, Shanghai Institute of Hematology, National Research Center for Translational Medicine, Shanghai Rui Jin Hospital, Shanghai Jiao Tong University School of Medicine, 197 Rui Jin Er Road, Shanghai, China

**Keywords:** Health services, Practice characteristics, Hematologists, Resources, China

## Abstract

**Background:**

In the past 40 years, China has experienced tremendous economic development, but the current situation of hematologists has rarely been reported. A landscape survey of human resources is essential for healthcare development and policy formulation in the future.

**Methods:**

The Chinese Society of Hematology initiated a survey of Chinese hematologists in mainland China for evaluating demographic and practice characteristics. Respondents were anonymous, and there were no limitations regarding their age, sex, etc.

**Results:**

Totally 2032 hematologists responded, with a median age bracket of 36–45 years. Respondents were well engaged into subspecialties, and 28.1% acquired doctorates of philosophy. Hematopoietic cell transplantation (HCT) centers have been established all over China. Higher-GDP regions reported more advantages, including bigger scale of transplant centers (*P* < 0.001), younger age structure (*P* = 0.039), better education qualifications (*P* = 0.001) and less turnover intentions (*P* = 0.004), despite of increased risk of medical disputes (*P* = 0.028). Although females accounted for 65.5% of hematologists, males were older (*P* < 0.001), and had more senior professional titles (*P* < 0.001), academic positions (*P* < 0.001), opportunities for continuing education (*P* < 0.001), and paper publishing in the recent two years (*P* = 0.001). For turnover intention, the higher GDP regions led to an independently reduced risk (HR = 0.673, 95%CI [0.482–0.940], *P* = 0.020), whereas medical disputes resulted in an increased the risk (HR = 2.037, 95%CI [1.513–2.743], *P* < 0.001). Considering the impact of the COVID-19 pandemic, majority of respondents believed that the decline in patient visits and delay in treatment was within 30%. 67.9% of respondents reported a decrease of the use of bone marrow as grafts but 18.8% reported an increase of cord blood units. 35.0% of the respondents switched their daily work to support the anti-epidemic medical activities.

**Conclusions:**

We concluded the discipline of hematology in China has flourished in recent years with a young workforce, while regional economic and gender disparities warrant further continuous optimization. Joint efforts against the impact of COVID-19 are needed in the post-pandemic era.

**Supplementary Information:**

The online version contains supplementary material available at 10.1186/s12913-024-10829-z.

## Background

Established in 1980, the Chinese Society of Hematology (CSH) developed rapidly and had original treatment protocols, such as the Beijing Protocol [1] or use of all-trans retinoic acid in the treatment of acute promyelocytic leukemia, [2] that have contributed to impressive achievements. Due to the high requirements for diagnostic platforms and laboratory conditions, hematological department tend to only exist in large hospitals. However, the current situation of health services of hematology in China, a developing country but also a rapidly developing economy for the last 40 years, has seldom been reported. A survey and investigation of the landscape of health services is a prerequisite for informed discussions on healthcare issues in China. Meanwhile, this survey might provide a reference for healthcare development and policy formulation in many other developing countries. Concerning the structure of hematologists especially practice in transplant patients is not quite clear, our survey focused on hematologists throughout China, with the aim of characterizing the distribution, structure, and practice of hematologists in hospitals in various regions of the country, as well as changes of transplant types and transplant delays during the ongoing coronavirus disease 2019 (COVID-19) pandemic.

The survey was designed by the CSH and the National Clinical Medical Research Centre for Hematologic Diseases (NCRCH) supported by the First Affiliated Hospital of Soochow University. A total of 31 provincial administrative regions (including provinces, municipalities and autonomous regions) in mainland China were included. Our survey included a series of questions exploring a variety of personal and professional characteristics.

## Methods

### Participants

A sample of 2,032 hematologists was assembled in this study from 31 provincial administrative regions throughout China by each provincial and then local municipal branch. To ensure adequate representation of hematologists, respondents were anonymous, and there were no limitations regarding age, sex, specialty, professional title, etc. The CSH committee reviewed the protocol and approved this study. The First Affiliated Hospital of Soochow University was commissioned to conduct the study.

### Study measures and data collection

The full-length survey included 32 questions collecting the basic demographic information of hematologists, clinical practice data and information on the impacts of the COVID-19 epidemic. The questionnaire used in this study was specifically developed for this survey and has not been published elsewhere before (See Supplement materials Questionnaire S5). Participants were not informed of the specific hypothesis of the study.

Questionnaires were administered using an online platform, namely, the Tencent questionnaire system (Version 2020), which accommodated smartphones, tablets, desktops and laptop PCs. The questionnaires were shared via a WeChat QR code and distributed by members of the CSH committee to the respective provincial hematological societies and municipal societies. Data collection was conducted between November 1st, 2020, and December 31st, 2020. All of the respondents were enrolled in the analysis.

### Definitions

The professional titles of physicians in China include four levels (junior, medium, vice-senior, and senior) certificated by provincial health administrative agencies (See supplement materials Table S1). The grade system of hospitals in China includes three levels (grade one, grade two, and grade three) in accordance with the current Administrative Measures for Hospital Grades. Grade 3 A represents the highest-level hospitals (See supplement materials Table S2). Higher-GDP and lower-GDP regions were categorized based on the median GDP per capita, which was calculated using the GDP per province by population per province in 2020 [3]. Multisited practice for physicians in China generally refers to the practice of healthcare professionals working or providing medical services in multiple locations or healthcare facilities including different hospitals, clinics, or community health centers.

### Statistical analysis

Standard descriptive statistics were used to characterize the responding hematologists. Baseline characteristics were compared by chi-square test. Univariate and multivariable analyses were performed using logistic regression, and all predictors with a *P* value below 0.10 in univariate analysis were included in multivariate analysis. All *P* values were two-sided and defined as statistically significant if the *P* value was less than 0.05. Statistical analyses were performed using SPSS 19.0 software (SPSS, Chicago, IL, USA) and the R 3.6.1 software package (The R Foundation for Statistical Computing, Vienna, Austria).

## Results

### Overview of the hematologists in China

**Fig. 1:**
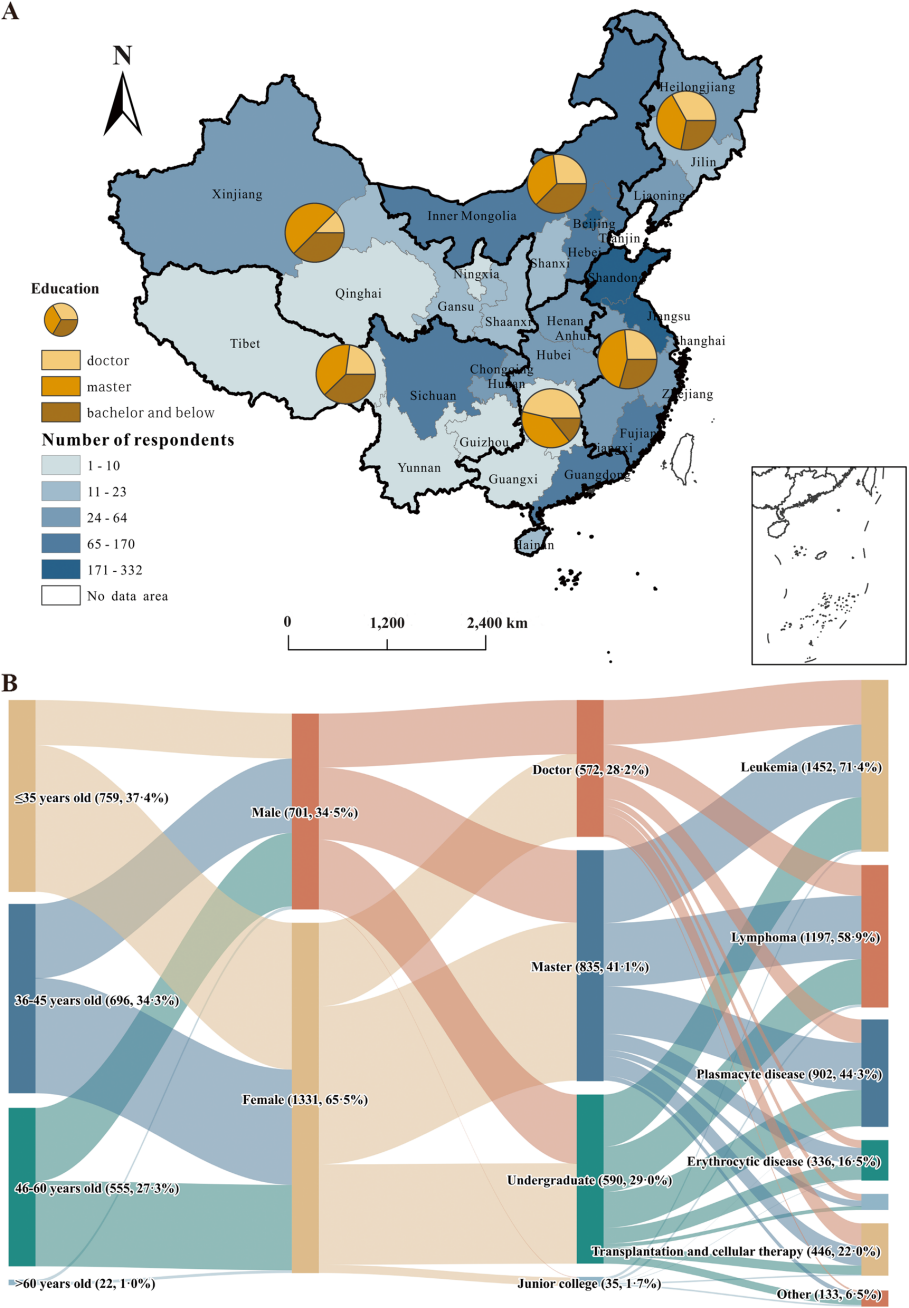
Demographic characteristics of the hematologists. **A **Distribution of the highest educational qualification in the six regions of mainland China; **B **Sankey diagram of demographic characteristics of hematologists

The questionnaire system showed that 4466 individuals received the questionnaire, of which 2032 respondents from 31 provincial administrative regions in mainland China submitted the full-length survey. Of the respondents, 701 (34.5%) were males. The median age bracket was 36–45, with 29.4% older than 45. Most hematologists obtained a master’s degree (835, 41.1%) or a bachelor’s degree (625, 30.8%), with less (572, 28.1%) holding a doctoral degree. According to the official classification of the National Bureau of Statistics of China, the country is geographically divided into six regions: North, Northeast, East, South Central, Southwest and Northwest [3]. The distribution of the qualification in six regions is shown in Fig. 1A. Figure 1B summarizes age, sex, qualifications, and subspecialties of the hematologists. Among the hematologists, most engaged in leukemia and lymphoma subspecialties (person-times: 1452 for leukemia, 1197 for lymphoma, 902 for plasmacyte disease, 336 for erythrocytic disease, 446 for transplantation and cellular therapy, 141 for thrombosis and hemostasis, and 135 for other subspecialties). The demographic and practical characteristics by regional economic level are summarized in Table 1.

**Table 1:** Characteristics of hematologists by regional economic level

		Overall (*n* = 2032)	Lower GDP regions (*n* = 1014)	Higher GDP regions (*n* = 1018)	*P* value
Geographic division	Northeast	115 (5.7%)	115 (11.3%)	0 (0.0%)	< 0.001
	North	467 (23.0%)	207 (20.4%)	260 (25.5%)	
	East	910 (44.8%)	356 (35.1%)	554 (54.4%)	
	Northwest	80 (3.9%)	80 (7.9%)	0 (0.0%)	
	Southwest	236 (11.6%)	190 (18.7%)	46 (4.5%)	
	South central	224 (11.0%)	66 (6.5%)	158 (15.5%)	
Transplant center scale	< 50 annually	916 (45.1%)	639 (63.0%)	277 (27.2%)	< 0.001
	≥ 50 annually	1116 (54.9%)	375 (37.0%)	741 (72.8%)	
Age	≤ 45	1455 (71.6%)	690 (68.0%)	765 (75.1%)	0.039
	>45	577 (28.4%)	324 (32.0%)	253 (24.9%)	
Sex	Female	1331 (65.5%)	681 (67.2%)	650 (63.9%)	0.117
	Male	701 (34.5%)	333 (32.8%)	368 (36.2%)	
Hospital grade	Non grade 3 A	244 (12.0%)	107 (10.6%)	137 (13.5%)	0.044
	Grade 3 A	1788 (88.0%)	907 (89.5%)	881 (86.5%)	
Type of work	Experimental research	100 (4.9%)	45 (4.4%)	55 (5.4%)	0.315
	Clinical practice	1932 (95.1%)	969 (95.6%)	963 (94.6%)	
Work experience	< 10 years	937 (46.1%)	407 (40.1%)	530 (52.1%)	< 0.001
	≥ 10 years	1095 (53.9%)	607 (59.9%)	488 (47.9%)	
Education	Doctor and master	1407 (69.2%)	666 (65.7%)	741 (72.8%)	0.001
	Undergraduate and below	625 (30.76%)	348 (34.3%)	277 (27.2%)	
Professional titles	Vice-senior and senior	986 (48.52%)	530 (52.3%)	456 (44.8%)	0.001
	Medium and below	1046 (51.5%)	484 (47.7%)	562 (55.2%)	
Hours of daily work	<8 h	297 (14.6%)	158 (15.6%)	139 (13.7%)	0.291
	8-10 h	1320 (65.0%)	654 (64.5%)	666 (65.4%)	
	11-14 h	352 (17.3%)	166 (16.4%)	186 (18.3%)	
	>14 h	63 (3.1%)	36 (3.6%)	27 (2.7%)	
Medical dispute	No	1704 (83.9%)	869 (85.7%)	835 (82.0%)	0.028
	Yes	328 (16.1%)	145 (14.3%)	183 (18.0%)	
Continuing education	yes	1057 (52.0%)	596 (58.8%)	461 (45.3%)	< 0.001
	no	975 (48.0%)	418 (41.2%)	557 (54.7%)	
Papers published in 2 years	No	925 (45.5%)	487 (48.0%)	438 (43.0%)	0.026
	Yes	1107 (54.5%)	527 (52.0%)	580 (57.0%)	
Multisited practice	No	1899 (93.5%)	975 (96.2%)	924 (90.8%)	< 0.001
	Yes	133 (6.6%)	39 ( 3.9%)	94 ( 9.2%)	
Turnover intention	No	1717 (84.5%)	833 (82.2%)	884 (86.8%)	0.004
	Yes	315 (15.5%)	181 (17.9%)	134 (13.2%)	

### Health services characteristics by region

Nine provinces were allocated to the higher-GDP regions, including 1018 respondents, while 22 provinces were allocated to the lower-GDP regions, including 1014 respondents (Table 1). There were no significant differences of hematologists between the higher- and lower-GDP regions in sex (*P* = 0.117), type of work (clinical practice or experimental research) (*P* = 0.315), or daily working time (*P* = 0.291). Although a bit more respondents from lower-GDP regions served in 3 A hospitals (89.5% versus 86.5%, *P* = 0.044) compared to higher-GDP regions, there were less served in large transplant centers (≥ 50 transplants annually) (37.0% versus 72.8%, *P* < 0.001). The age structure of hematologists in higher-GDP regions appeared to be younger (*P* = 0.039), with 75.1% under the age of 45 compared to 68.0% in lower-GDP regions. Accordingly, the proportions of hematologists with ≥ 10 years of experience (59.9% versus 47.9%, *P* < 0.001) and senior titles (52.3% versus 44.8%, *P* = 0.001) in lower-GDP regions were higher compared to higher-GDP regions. Nevertheless, 9 provinces in higher-GDP regions had 38 positions on the CSH committee, while the other 22 provinces had 33 positions. In addition, respondents from higher-GDP regions had better educational qualifications (doctoral degree or master’s degree) than those from lower-GDP regions (72.8% versus 65.7%, *P* = 0.001). Respondents from lower-GDP regions had more continuing education experience (58.8% versus 45.3%, *P* < 0.001). A total of 57.0% of respondents in higher-GDP regions had published at least one scientific paper in recent 2 years, compared to 52.0% in lower-GDP regions (*P* = 0.026). More respondents from higher-GDP regions engaged in multisided practice (9.2% versus 3.9%, *P* < 0.001), and fewer had turnover intention (13.2% versus 17.9%, *P* = 0.004). Interestingly, fewer respondents reported medical disputes in lower-GDP regions than in higher-GDP regions (14.3% versus 18.0%, *P* = 0.028).

### Practical characteristics of hematologists by sex

Differences in practical characteristics could also be observed between male and female hematologists (Table 2). Generally, males were older (> 45 years old: 37.5% versus 23.6%, *P* < 0.001) and had more work experience (62.6% versus 49.3%, *P* < 0.001). Regarding their professional careers, males might be less likely to have a doctoral degree or master’s degree (66.5% versus 70.7%, *P* = 0.050) but more likely to have senior professional titles (60.6% versus 42.1%, *P* < 0.001) as well as national and provincial academic positions (55.3% versus 39.0%, *P* < 0.001). In addition, males had more opportunities for continuing education experience (61.8% versus 46.9%, *P* < 0.001) and paper publishing (59.3% versus 51.9%, *P* = 0.001). The frequency of multisided practice (9.0% versus 5.3%, *P* = 0.001) and medical disputes (21.0% versus 13.6%, *P* < 0.001) was also higher in males than in females.

**Table 2:** Characteristics of hematologists by sex

		Overall (*n* = 2032)	Male (*n* = 701)	Female (*n* = 1331)	*P* value
Geographic division	Northeast	115 (5.7%)	29 (4.1%)	86 (6.5%)	< 0.001
	North	467 (23.0%)	110 (15.7%)	357 (26.8%)	
	East	910 (44.8%)	362 (51.6%)	548 (41.2%)	
	Northwest	80 (3.9%)	36 (5.1%)	44 (3.3%)	
	Southwest	236 (11.6%)	86 (12.3%)	150 (11.3%)	
	South central	224 (11.0%)	78 (11.1%)	146 (11.0%)	
Transplant center scale	< 50 annually	916 (45.1%)	331 (47.2%)	585 (44.0%)	0.160
	≥ 50 annually	1116 (54.9%)	370 (52.8%)	746 (56.0%)	
Age	≤ 45	1455 (71.60%)	438 (62.5%)	1017 (76.4%)	< 0.001
	>45	577 (28.40)	263 (37.5%)	314 (23.6%)	
Hospital grade	Grade 3 A	244 (12.0%)	110 (15.7%)	134 (10.1%)	< 0.001
	Non grade 3 A	1788 (88.0%)	591 (84.3%)	1197 (89.9%)	
Type of work	Experimental research	100 (4.9%)	30 (4.3%)	70 (5.3%)	0.332
	Clinical practice	1932 (95.1%)	671 (95.7%)	1261 (94.7%)	
Work experience	< 10 years	937 (46.11%)	262 (37.4%)	675 (50.7%)	< 0.001
	≥ 10 years	1095 (53.89%)	439(62.6%)	656 (49.3%)	
Education	Doctor and master	1407 (69.24%)	466 (66.5%)	941 (70.7%)	0.050
	Undergraduate and below	625 (30.76%)	235 (33.5%)	390 (29.3%)	
Professional title	Vice-senior and senior	986 (48.52%)	425 (60.6%)	561 (42.1%)	< 0.001
	Medium and below	1046 (51.48%)	276 (39.4%)	770 (57.9%)	
Academic position	National and provincial academic position	907 (44.64%)	388 (55.3%)	519 (39.0%)	< 0.001
	Municipal or none	1125 (55.36%)	313(44.7%)	812 (61.0%)	
Hours of daily work	<8 h	297 (14.6%)	98 (14.0%)	199 (15.0%)	0.425
	8-10 h	1320 (65.0%)	471 (67.2%)	849 (63.8%)	
	11-14 h	352 (17.3%)	114 (16.3%)	238 (17.9%)	
	>14 h	63 (3.1%)	18 (2.6%)	45 (3.4%)	
Medical dispute	No	1704 (83.9%)	554 (79.0%)	1150 (86.4%)	< 0.001
	Yes	328 (16.1%)	147 (21.0%)	181 (13.6%)	
Continuing education	yes	1057 (52.0%)	433 (61.8%)	624 (46.9%)	< 0.001
	no	975 (48.0%)	268 (38.2%)	707 (53.1%)	
Papers published in 2 years	No	925 (45.5%)	285 (40.7%)	640 (48.1%)	0.001
	Yes	1107 (54.5%)	416 (59.3%)	691 (51.9%)	
Multisited practice	No	1899 (93.5%)	638 (91.0%)	1261 (94.7%)	0.001
	Yes	133 (6.5%)	63 (9.0%)	70 (5.3%)	
GDP region	Lower	1014 (49.9%)	333 (47.5%)	681 (51.2%)	0.117
	Higher	1018 (50.1%)	368 (52.5%)	650 (48.8%)	
Turnover intention	No	1717 (84.5%)	587 (83.7%)	1130 (84.9%)	0.492
	Yes	315 (15.5%)	114 (16.3%)	201 (15.1%)	

### Subspecialties for hematologic services

Hematologic services were well subdivided into subspecialties according to our survey. Because an individual may be engaged in more than one subspecialty, the question of subspecialty in the survey was multiple choice and measured by person-times. More than half of the hematologists who engaged in the subspecialties of leukemia (53.0%), lymphoma (54.6%), plasmacyte disease (58.2%), erythrocytic disease (55.4%), and thrombosis and hemostasis (54.6%) had experience with continuing studies, while less than half who engaged in the transplantation and cellular therapy (49.3%) subspecialty, and much fewer (43.0%) who engaged in other subspecialties (congenital disease, etc.) had experience in continuing studies (*P* < 0.001). More hematologists who engaged in the leukemia (55.0%), lymphoma (54.0%), plasmacyte disease (56.8%), thrombosis and hemostasis (63.8%), and transplantation and cellular therapy (61.4%) subspecialties had published papers in the last two years, while the percentage was lower for the erythrocytic disease subspecialty (48.8%) and other subspecialties (28.3%) (*P* < 0.001). Apart from the above, sex, daily work time, and turnover intention were statistically comparable among these subspecialties (Table S3 in the Supplement).

### Turnover intention and medical disputes

In aggregate, 15.5% respondents reported turnover intention. Variables associated with turnover intention identified by univariate analysis included GDP region (*P* = 0.004), large transplant centers (*P* = 0.047), age (*P* = 0.001), grade of the hospital (*P* = 0.056), professional title (*P* < 0.001), hours of daily work (*P* = 0.013), medical disputes (*P* < 0.001), and continuing education (*P* = 0.011) (Table S4 in the Supplement). Multivariate analysis showed that higher-GDP regions had an independently reduced risk of turnover intention (HR = 0.673, 95% CI [0.482–0.940], *P* = 0.020), whereas medical disputes were related to an increased risk (HR = 2.037, 95% CI [1.513–2.743], *P* < 0.001). In addition, professional title was marginally associated with turnover attention (*P* = 0.050) (Table 3).

Doctor–patient disputes were further investigated considering their significant impact on turnover intention in respondents. The results of comparisons revealed that respondents from higher-GDP regions (18.0% versus 14.3%, *P* = 0.028), those from non-Grade 3 A hospitals (21.3% versus 15.4%, *P* = 0.025) and male respondents (21.0% versus 13.6%, *P* < 0.001) had more disputes, while they were comparable among respondents with different ages (*P* = 0.200), professional titles (*P* = 0.277), hours of daily work (*P* = 0.355) and educational qualifications (*P* = 0.658).

**Table 3:** Multivariate analysis for turnover intention

		OR (95%CI)	P
GDP region	Higher	0.673 (0.482–0.940)	0.020
Transplant center scale^a^	Transplant center scale	NA	0.763
Age			0.075
	≤ 35	1	
	36–45	1.272 (0.972–1.856)	0.212
	46–60	0.749 (0.433–1.295)	0.301
	>60	0	0.998
Hospital grade	Non grade 3 A	0.800 (0.556–1.152)	0.231
Professional title			0.050
	Senior	1	
	Vice-senior	1.060 (0.663–1.695)	0.808
	Medium	1.427 (0.831–2.452)	0.198
	Junior and below	0.885 (0.451–1.735)	0.722
Hours of daily work			0.062
	<8 h	1	
	8–10 h	1.284 (0.867–1.902)	0.212
	11–14 h	1.483 (0.938–2.345)	0.092
	>14 h	2.421 (1.513–2.743)	0.011
Medical dispute	Yes	2.037 (1.513–2.743)	< 0.001
Continuing education			0.124
	Abroad	1	
	Domestic	1.572 (0.935–2.643)	0.088
	Both	1.039 (0.466–2.313)	0.926
	Never	1.169 (0.67502.027)	0.577

^a^Transplant center scale is a continuous variable here, which is measured by HSCT cases per year in a transplant center. The data is from the CSH

### Impacts of the epidemic on health services

**Fig. 2:**
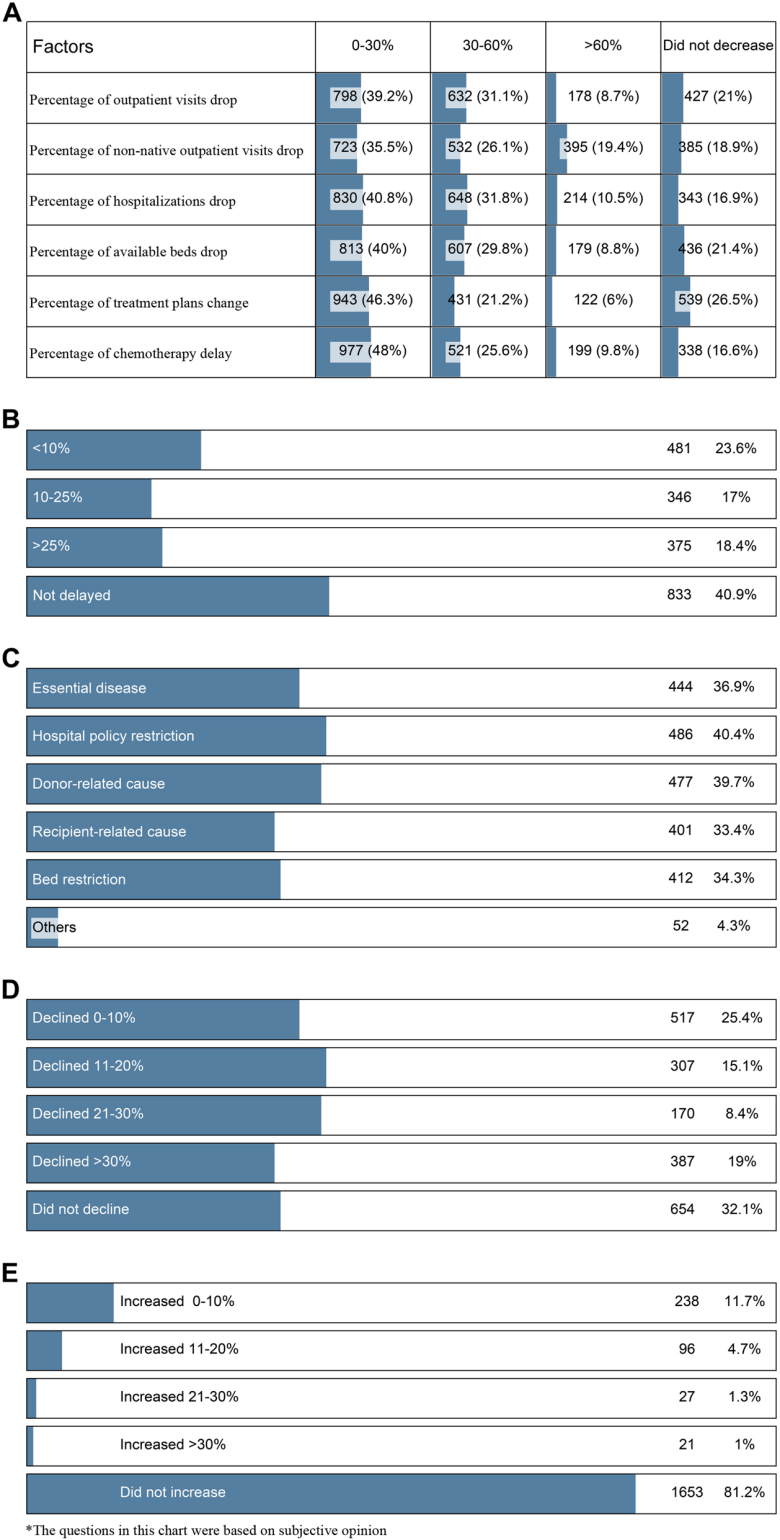
Subjective impacts of the epidemic on the activities of hematologists. **A **Decline and delay of treatment due to the epidemic; **B **Percentage of auto-HSCT delay; **C **Main reasons for allo-HSCT delay; **D **Reduction in the use of bone marrow as a graft in allo-HSCT; **E** Increase in the use of umbilical cord blood in allo-HSCT

 In terms of the decline in outpatient visits, 39.2% of respondents believed that outpatient visits had dropped by less than 30%, 31.1% of respondents believed that they had dropped by 30-60%, and 8.7% believed that they had dropped by more than 60%, while 21% of respondents thought that had not declined. Regarding visits by non-native patients, 35.5% believed that outpatient visits had dropped by less than 30%, 26.1% of the respondents believed that they had dropped by 30-60%, 19.4% believed that they had dropped by more than 60%, and 18.9% thought that they had not declined. For hospitalizations, 40.8% of respondents believed that they had dropped by less than 30%, 31.8% of respondents believed that they had dropped by 30-60%, 10.5% believed that they had dropped by more than 60%, and 16.9% of respondents thought that not declined. Regarding patients changing treatment plans, nearly half (46.3%) of the respondents believed that less than 30% of patients changed treatment plans, 21.2% believed that 30-60% patients changed treatment plans, 6% believed that more than 60% patients changed treatment plans, and 26.5% said no change. For delayed chemotherapy, approximately half respondents (48.0%) believed that less than 30% of patients changed treatment plans, 25.6% believed that 30-60% patients changed, and 9.8% believed that more than 60% patients changed, but 16.6% said that there was no delay (Fig. 2A).

Comparing the initial most serious period of the epidemic (January-April 2019) with the same period of the following year (January-April 2020), 40.9% of the respondents believed that autologous hematopoietic stem cell transplantation (auto-HSCT) had not been delayed, while 23.6% believed that within 10% had been delayed, 17.0% believed that 10-25% had been delayed, and 18.4% believed more than 25% (Fig. 2B). Concerning the main reasons for allogeneic hematopoietic stem cell transplantation (allo-HSCT) delay, the options in order of most to least common were as follows: hospital policy restriction (40.4%), donor-related cause (39.7%), essential disease (36.9%), bed restriction (34.3%), recipient-related cause (33.4%), and others (4.3%) (Fig. 2C). In terms of the reduction in bone marrow use as a graft in allo-HSCT, 32.1% of the respondents believed that it had not declined, 25.4% said that it had dropped by less than 10%, 15.1% believed that it had declined 11-20%, 8.4% believed that it had declined 21-30%, and 19% said that the reduction rate exceeded 30% (Fig. 2D). The vast majority (81.2%) of respondents believed that there was no increase in the use of umbilical cord blood in allo-HSCT; 11.7% of the respondents believed that the increase rate was less than 10%, 4.7% believed that the use had increased by 11-20%, 1.3% believed that it had increased by 21-30%, and 1% believed more than 30% (Fig. 2E).

During the epidemic, 35.0% of the respondents temporarily switched their work to fight against COVID-19 in different ways. Among them, 71.4% switched their posts to fever clinics, 13.7% to pretest triage, 2.3% to diagnostic laboratory, and 12.5% to other posts. Respondents with medium professional titles had the highest proportion of work switch (44.5%), followed by those with vice-senior titles (36.0%), junior titles (33.2%) and senior titles (22.4%, *P* < 0.001).

## Discussion

China’s economic growth over the past four decades has generated unprecedented healthcare conditions, including in hematology. In recent decades, hematology has developed rapidly in China, but there is a lack of studies depicting the landscape of hematologic health services in China. This is the first nationwide report of Chinese hematologists evaluating health services and practical characteristics. The results reported herein are intended to provide a broader vision to facilitate a better understanding of the current situation of hematologic health services in the developing world and support decision making in the future. Since there are few similar published reports, we supposed our experience might also favor the development of hematologists in other developing countries.

A total of 2032 hematologists from 31 provincial administrative regions responded. Based on the submitted questionnaires, we performed a comprehensive analysis involving individual characteristics, regional differences, practical patterns and challenges derived from the epidemic for the Chinese hematologists’ workforce. There were many differences in the characteristics of hematologists by region and sex, as well as the impactors of turnover intention and the current state of medical disputes under contemporary medical circumstances. In addition, the influences of the epidemic on therapeutic health services were also analyzed.

Due to the complexity of hematological diseases, hematologic services heavily rely on a well-equipped laboratory platform and, more importantly, highly educated hematologists with a strong capacity for continuing study. In our investigation, approximately 70% of hematologists in China were younger than 45. The proportion of higher educational qualifications among young physicians was significantly higher than that of older physicians, indicating that research capability of Chinese hematologists is improving. Although there was a higher proportion of males with senior titles and academic appointments, it was noted that there was an increasing number of highly educated females among the younger respondents. A series of studies revealed sex inequality in clinical disciplines, including oncology, [4] cardiology, [5] gastroenterology, [6] and radiology, [7, 8] for various reasons [9–12]. As a developing country, the proportion of newly licensed female physicians in China rose from 51% in 2005 to 56% in 2015, [13] higher than previously reported data in U.S [14]. and Canada [15]. However, it was shown in our study that female hematologists seemed to get less occasions for the development of career. The development of hematologic health services is affected by the local economic level. It has been reported that medical workers from higher-GDP regions have more social resources, [16–21] which is in accordance with our results. However, in our study, respondents from lower-GDP regions were more likely to be from Grade 3 A hospitals. A potential reason is that most hematological departments can only exist in top-level hospitals in these regions, which can afford expensive laboratory facilities and transplant units [22–24]. The reason why respondents from the lower-GDP regions had more continuing education was probably that respondents from the higher-GDP regions could continue studying in their own workplaces. In addition, the distribution of academic appointments and research ability were significantly unbalanced between higher- and lower-GDP regions in our survey. Disparities in healthcare are a recognized issue in China and other developing countries, and efforts should be taken to minimize their impact on treatment outcomes. Even developed countries with established universal health insurance systems also face challenges. For instance, it was reported that rural area tended to have higher disease mortality in the treatment of leukemia in the US, although not supported by the Japanese data [25, 26]. The lack of resources and voices in less developed regions should be taken into account for healthcare policy in the future. The employed methods in lagging areas included expanding the team of medical workers, supporting the deployment of advanced techniques and devices, strengthening continuing education and training, establishing and popularizing adapted clinical pathways and applicable guidelines, and developing telemedicine and intelligent medicine, etc. It is important to encourage young and well-educated physicians to work in various regions and ensure a more equitable presence of hospitals.

Medical disputes emerged as the strongest predictor of turnover intention in our study, which was also a focal point in other studies [27–29]. Therefore, both in developed and developing countries, it is necessary to implement various initiatives to promote harmonious doctor-patient relations, which can help reduce turnover intention of physicians. A higher-GDP level also independently reduced turnover intention, probably because of a satisfactory salary and social resources [30]. Intriguingly, the lower-GDP regions had fewer medical disputes. Contrary to the findings that workload increased burnout symptoms and turnover intention in other studies, [31, 32] the daily working time (similar to the U.S. oncologists [32]) had no impact on turnover intention in our study.

Regarding the impact of the epidemic on health services, the majority of respondents believed that the decline in patient visits and delay in treatment were within 30%. The lockdown policy possibly contributed to the negative impacts on clinical practice. On the other hand, government support measures minimized the impacts on healthcare system, although the majority of respondents (72.9%) reported a decrease in wages during the outbreak. Regarding the impact on transplantation, a number of respondents reported a decrease in the use of bone marrow but an increase in the use of cord blood, consistent with the 2020 annual report of the China Bone Marrow Transplant Registry (CBMTR). The number of cord blood transplant cases increased by 57 cases compared to 2019, reaching 571 cases. This might be related to the storage of umbilical cord blood units, which could be taken immediately and avoid the risk related to donors and graft collection procedures. According to the data from the CBMTR, the annual number of transplants in 2020 (*n* = 13,415) was 8.8% lower than that in 2019 (*n* = 12,323), which hinted the negative impact of the epidemic. It deserves further thinking on how to maintain HSCT practice during a pandemic.

The limitations of this study include the inability to recruit all the Chinese hematologists, the imbalanced response rate among different provinces, and the lack of concrete data for further analysis. Nevertheless, the sample size of demographic data among other surveys of physicians is similar to ours [31, 33, 34]. Additionally, the age distribution of the population in our survey is consistent with that of practicing physicians in China, as reported in the “China Statistical Yearbook of Health 2021” by the National Health Commission of the People’s Republic of China [35].

## Conclusion

In the background of the flourished hematologic health services in recent years, the hematologist workforce continues to progress in China. However, the regional and gender disparities, which led to burnout and turnover intention of hematologists in underdeveloped regions, warranted further optimization. We suggested that medical resources should be tilted towards underdeveloped regions, meanwhile strengthening the support for the careers of local hematologists. It is hoped that this report will provide a reference for developing countries to further formulate health policies.

### Supplementary Information

**Supplementary Material 1.**

**Supplementary Material 2.**

**Supplementary Material 3.**

**Supplementary Material 4.**

**Supplementary Material 5.**

## Data Availability

The datasets generated and/or analysed during the current study are not publicly available due to the large number of questionnaires and the limitation of excessive data volume but are available from the corresponding author on reasonable request.
