# Supplementary material for: Hematologic health services and practical characteristics: report of a nationwide survey among Chinese hematologists
Source: BMC Health Serv Res. 2024 Mar 12;24:326. doi: 10.1186/s12913-024-10829-z (PMC10929140; doi:10.1186/s12913-024-10829-z)
Supplement: Supplementary file 1 — Supplementary Material 1. [file 12913_2024_10829_MOESM1_ESM.docx]

**Table S1 Physician Ranking System**

| Position | Rank | Description |
| --- | --- | --- |
| Resident Physician | Junior | Junior doctors undergoing practical training and gaining hands-on experience in hospitals. |
| Attending Physician | Medium | Licensed doctors with full responsibility for patient care, having completed their residency. |
| Associate Chief Physician | Vice-senior | Doctors with substantial experience, taking on leadership and complex medical roles. |
| Chief Physician | Senior | The most experienced doctors usually leading a clinical team, involved in advanced patient care, teaching, and research. |
