# Supplementary material for: Hematologic health services and practical characteristics: report of a nationwide survey among Chinese hematologists
Source: BMC Health Serv Res. 2024 Mar 12;24:326. doi: 10.1186/s12913-024-10829-z (PMC10929140; doi:10.1186/s12913-024-10829-z)
Supplement: Supplementary file 2 — Supplementary Material 2. [file 12913_2024_10829_MOESM2_ESM.docx]

**Table S2 Grading System for Hospitals in China**

| Hospital Level | Description |
| --- | --- |
| Primary Hospitals | Basic healthcare clinics, providing disease prevention service, primary medical care, and rehabilitation usually in a community. |
| Secondary Hospitals | Bigger healthcare facilities possess internal medical and surgical departments, which providing comprehensive medical care usually for a town. |
| Tertiary Hospitals | The highest level of hospitals in China, including the sub-levels of 3A (top), 3B, and 3C. |
| - 3A Hospitals | Usually a hospital affiliated to a medical university or college, which provide high-quality medical care including emergent or critical care service. They possess complete departments even with sub-specialties, sufficient equipments and emerging technologies. |
