# Supplementary material for: Hematologic health services and practical characteristics: report of a nationwide survey among Chinese hematologists
Source: BMC Health Serv Res. 2024 Mar 12;24:326. doi: 10.1186/s12913-024-10829-z (PMC10929140; doi:10.1186/s12913-024-10829-z)
Supplement: Supplementary file 3 — Supplementary Material 3. [file 12913_2024_10829_MOESM3_ESM.docx]

|  |  | Leukaemia | Lymphoma | Plasmacyte disease | Erythrocytic disease | Thrombosis and haemostasis | Transplantation and cellular therapy | Other subspecialties | P value |
| --- | --- | --- | --- | --- | --- | --- | --- | --- | --- |
|  | Total (n = 2032) | 1452 | 1197 | 902 | 336 | 141 | 446 | 135 |  |
| Sex | Male | 503（34.6%） | 417（34.8%） | 318（35.3%） | 134（39.9%） | 59（41.8%） | 179（40.1%） | 44（32.6%） | 0.111 |
|  | Female | 949（65.4%） | 780（65.2%） | 584（64.7%） | 202（60.1%） | 82（52.2%） | 267（59.9%） | 91（67.4%） |  |
| Hours of daily work | ≤10h | 1165（80.2%） | 959（80.1%） | 738（81.8%） | 258（76.8%） | 111（78.7%） | 337（75.6%） | 107（79.3%） | 0.148 |
|  | ＞10h | 287（19.8%） | 238（19.9%） | 164（18.2%） | 78（23.2%） | 30（21.3%） | 109（24.4%） | 28（20.7%） |  |
| Continuing education | Yes | 770（53.0%） | 653（54.6%） | 525（58.2%） | 186（55.4%） | 77（54.6%） | 220（49.3%） | 58（43.0%） | < 0.001 |
|  | No | 682（47.0%） | 544（45.4%） | 377（41.8%） | 150（44.6%） | 64（45.4%） | 226（50.7%） | 77（57.0%） |  |
| Paper published in 2 years | Yes | 799（55.0%） | 646（54.0%） | 512（56.8%） | 164（48.8%） | 90（63.8%） | 274（61.4%） | 38（28.1%） | < 0.001 |
|  | No | 653（45.0%） | 551（46.0%） | 390（43.2%） | 172（51.2%） | 51（36.2%） | 172（38.6%） | 97（71.9%） |  |
| Turnover intention | Yes | 240（16.5%） | 190（15.9%） | 150（16.6%） | 55（16.4%） | 18（12.8%） | 60（13.5%） | 19（14.1%） | 0.647 |
|  | No | 1212（83.5%） | 1007（84.1%） | 752（83.4%） | 281（83.6%） | 123（87.2%） | 386（86.5%） | 116（85.9%） |  |

**Table S3 Analysis for subspecialties of haematology**
