# Supplementary material for: Hematologic health services and practical characteristics: report of a nationwide survey among Chinese hematologists
Source: BMC Health Serv Res. 2024 Mar 12;24:326. doi: 10.1186/s12913-024-10829-z (PMC10929140; doi:10.1186/s12913-024-10829-z)
Supplement: Supplementary file 4 — Supplementary Material 4. [file 12913_2024_10829_MOESM4_ESM.doc]

Table S4 Univariate analysis for turnover intention

|  |  |  | **Turnover intention** | | **OR (95%CI)** | **P value** |
| --- | --- | --- | --- | --- | --- | --- |
|  |  |  | **Yes** | **No** |  |  |
| GDP region | Lower | 1014 (49.9%) | 181 (57.5%) | 833 (48.5%) | 1 |  |
|  | Higher | 1018 (50.1%) | 134 (42.5%) | 884 (51.5%) | 0.698 (0.547 – 0.889) | 0.004 |
| Geographic division |  |  |  |  |  | 0.156 |
|  | Northeast | 115 (5.7%) | 19 (6.0%) | 96 (5.6%) | 1 |  |
|  | North | 467 (23.0%) | 77 (24.4%) | 390 (22.7%) | 0.998 (0.576 – 1.728) | 0.993 |
|  | East | 910 (44.8%) | 139 (44.1%) | 771 (44.9%) | 0.911 (0.539 – 1.539) | 0.727 |
|  | Northwest | 80 (3.9%) | 20 (6.3%) | 60 (3.5%) | 1.684 (0.831 – 3.412) | 0.148 |
|  | Southwest | 236 (11.6%) | 32 (10.2%) | 204 (11.9%) | 0.793 (0.428 – 1.469) | 0.460 |
|  | South central | 224 (11.0%) | 28 (8.9%) | 196 (11.4%) | 0.722 (0.384 –1.358) | 0.312 |
| Transplant centre scale* |  | 2032 (100%) | 315 (15.5%) | 1717 (84.5%) | NA | 0.047 |
| Age |  |  |  |  |  | 0.001 |
|  | ≤35 | 759 (37.4%) | 109 (34.6%) | 650 (37.9%) | 1 |  |
|  | 36-45 | 696 (34.3%) | 140 (44.4%) | 556 (32.4%) | 1.502 (1.141 – 1.976) | 0.004 |
|  | 46-60 | 555 (27.3%) | 66 (21.0%) | 489 (28.5%) | 0.805 (0.580 – 1.117) | 0.194 |
|  | ＞60 | 22 (1.1%) | 0 (0.0%) | 22 (1.3%) | 0.000 | 0.998 |
| Sex | Female | 1331 (65.5%) | 201 (63.8%) | 1130 (65.8%) | 1 |  |
|  | Male | 701 (34.5%) | 114 (36.2%) | 587 (34.2%) | 1.092 (0.850 – 1.403) | 0.492 |
| Hospital grade | Grade 3A | 244 (12.0%) | 48 (15.2%) | 196 (11.4%) | 1 |  |
|  | Non grade 3A | 1788 (88.0%) | 267 (84.8%) | 1521 (88.6%) | 0.717 (0.509 – 1.008) | 0.056 |
| Type of work | Experimental research | 100 (4.9%) | 10 (3.2%) | 90 (5.2%) | 1 |  |
|  | Clinical practice | 1932 (95.1%) | 305 (96.8%) | 1627 (94.8%) | 1.687 (0.868 – 3.280) | 0.123 |
| Working experience |  |  |  |  |  | 0.168 |
|  | ＜3 years | 295 (14.5%) | 37 (11.7%) | 258 (15.0%) | 1 |  |
|  | 3-5 years | 274 (13.5%) | 36 (11.4%) | 238 (13.9%) | 1.055 (0.645 – 1.724) | 0.832 |
|  | 6-10 years | 368 (18.1%) | 66 (21.0%) | 302 (17.6%) | 1.524 (0.986 – 2.355) | 0.058 |
|  | ＞10 years | 1095 (53.9%) | 176 (55.9%) | 919 (53.5%) | 1.335 (0.913 – 1.954) | 0.136 |
| Education |  |  |  |  |  | 0.408 |
|  | Doctor | 572 (28.1%) | 82 (26.0%) | 490 (28.5%) | 1 |  |
|  | Master | 835 (41.1%) | 140 (44.4%) | 695 (40.5%) | 1.204 (0.895 – 1.618) | 0.220 |
|  | Undergraduate and below below | 625 (30.8%) | 93 (29.5%) | 532 (31.0%) | 1.045 (0.758 – 1.440) | 0.790 |
| Professional title |  |  |  |  |  | < 0.001 |
|  | Senior | 455 (22.4%) | 51 (16.2%) | 404 (23.5%) | 1 |  |
|  | Vice-senior | 531 (26.1%) | 89 (28.3%) | 442 (25.7%) | 1.595 (1.102 – 2.309) | 0.013 |
|  | Medium | 636 (31.3%) | 129 (41.0%) | 507 (29.5%) | 2.016 (1.421 – 2.859) | < 0.001 |
|  | Junior and below | 410 (20.2%) | 46 (14.6%) | 364 (21.2%) | 1.001 (0.656 – 1.528) | 0.996 |
| Academic positions | No | 883 (43.5%) | 132 (41.9%) | 751 (43.7%) | 1.078 (0.845 – 1.375) | 0.546 |
|  | Yes | 1149 (56.5%) | 183 (58.1%) | 966 (56.3%) |  |  |
|  |  |  |  |  |  | 0.013 |
| Hours of daily work | ＜8h | 297 (14.6%) | 35 (11.1%) | 262 (15.3%) | 1 |  |
|  | 8-10h | 1320 (65.0%) | 200 (63.5%) | 1120 (65.2%) | 1.337 (0.911 – 1.961) | 0.138 |
|  | 11-14h | 352 (17.3%) | 63 (20.0%) | 289 (16.8%) | 1.632 (1.045 – 2.548) | 0.031 |
|  | ＞14h | 63 ( 3.1%) | 17 ( 5.4%) | 46 ( 2.7%) | 2.766 (1.432 – 5.346) | 0.002 |
| Medical dispute | No | 1704 (83.9%) | 237 (75.2%) | 1467 (85.4%) | 1 |  |
|  | Yes | 328 (16.1%) | 78 (24.8%) | 250 (14.6%) | 1.931 (1.447 – 2.578) | < 0.001 |
| Continuing education |  |  |  |  |  | 0.011 |
|  | Abroad | 189 (9.3%) | 20 (6.3%) | 169 (9.8%) | 1 |  |
|  | Domestic | 778 (38.3%) | 145 (46.0%) | 633 (36.9%) | 1.936 (1.177 – 3.183) | 0.009 |
|  | Both | 90 (4.4%) | 11 (3.5%) | 79 (4.6%) | 1.177 (0.538 – 2.574) | 0.684 |
|  | Never | 975 (48.0%) | 139 (44.1%) | 836 (48.7%) | 1.405 (0.855 – 2.310) | 0.180 |
| Papers in 2 years | No | 925 (45.5%) | 138 (43.8%) | 787 (45.8%) | 1 |  |
|  | Yes | 1107 (54.5%) | 177 (56.2%) | 930 (54.2%) | 1.085 (0.852 – 1.383) | 0.507 |
| Multisited practice | No | 1899 (93.5%) | 288 (91.4%) | 1611 (93.8%) | 1 |  |
|  | Yes | 133 (6.5%) | 27 (8.6%) | 106 (6.2%) | 1.425 (0.917 – 2.214) | 0.115 |

*Transplant centre scale is a continuous variable here, which is measured by HSCT cases annually in a transplant centre. The data is from the CSH.
