# Supplementary material for: Hematologic health services and practical characteristics: report of a nationwide survey among Chinese hematologists
Source: BMC Health Serv Res. 2024 Mar 12;24:326. doi: 10.1186/s12913-024-10829-z (PMC10929140; doi:10.1186/s12913-024-10829-z)
Supplement: Supplementary file 5 — Supplementary Material 5. [file 12913_2024_10829_MOESM5_ESM.docx]

the Second Nationwide Survey among Chinese Hematologists

During the COVID-19 pandemic control period, grassroots medical and health institutions and medical staffs played a significant role. This survey aims to understand the situation of hematologists nationwide in terms of work undertaken during the pandemic, which is expected to serve as an important basis for our plan formulation. This survey questionnaire consists of 32 questions and is estimated to take 7 minutes to complete.

Part One: Personal Information [Single-choice Questions] [Mandatory]

(Your age:)

≤35 years old

36-45 years old

46-60 years old

＞60 years old

(Your sex:) [Single-choice Questions] [Mandatory]

Male

Female

(Your current position in the hospital:) [Single-choice Questions] [Mandatory]

Permanent staff

Contractual Employee

Temporary Staff

(The grade of your hospital:) [Single-choice Questions] [Mandatory]

Tertiary Hospitals（3A）

Tertiary Hospitals（3B）

Secondary Hospitals（3A）

Secondary Hospitals（3B）

Primary Hospitals

(Your hospitals’ geographic division:) _________; The name of your hospital: _________; The type of your hospital is: [Single-choice Question] [Mandatory]

National

Provincial

Municipal

County

Private

Military Hospital

Other____

(Your type of work:) [Single-choice Questions] [Mandatory]

Clinical practice

Laboratory diagnosis

Other____

(Did you switch your work to fight against COVID-19) [Single-choice Questions] [Mandatory]

Laboratory technician

Fever clinics

Pretesting triage

No change

Other____

(How long have you been working in this hospital?) [Single-choice Questions] [Mandatory]

Less than 3 years

3 to 5 years

6 to 10 years

10 years or more

(What is your educational background:) [Single-choice Questions] [Mandatory]

Doctoral degree

Master's degree

Associate degree

Technical secondary school and below

(What is your professional title:) [Single-choice Questions] [Mandatory]

Senior professional title

Vice-senior professional title

Medium professional title

Junior or below

Residency

Internship

(Do you hold any academic positions?) [Multiple-choice Question] [Mandatory]

National Medical Academic Committee Chairman

National Medical Academic Committee Vice Chairman

National Medical Academic Committee Standing Committee Member

National Medical Academic Committee Member

Provincial Medical Academic Committee Chairman

Provincial Medical Academic Committee Vice Chairman

Provincial Medical Academic Committee Member

Municipal Medical Academic Committee Chairman

Municipal Medical Academic Committee Vice Chairman

Municipal Medical Academic Committee Member

None of the above

(What was your average time of daily work before the pandemic?) [Single-choice Questions] [Mandatory]

Less than 8 hours

8 to 10 hours

11 to 14 hours

More than 14 hours

(Did your working hours change during the pandemic (January-April 2020)) [Single-choice Questions] [Mandatory]

No change

Extended working hours

Reduced working hours

(Did your income change during the pandemic?) [Single-choice Questions] [Mandatory]

No change

Increased income

Decreased income

(In the past year, have you had any medical disputes?) [Single-choice Questions] [Mandatory]

Never

5 or fewer

6 to 10

11 to 20

More than 20

(Did the pandemic delay your continuing education?) [Single-choice Questions] [Mandatory]

Yes

No

(Do you have any experience in continuing education?) [Single-choice Questions] [Mandatory]

Overseas continuing education

Domestic continuing education

Both domestic and overseas continuing education

No further education experience

(Which domestic hospital did you participate in for continuing education (for those who further educated domestically)) [Single-choice Questions] [Mandatory]

National Key Discipline/Specialty/Laboratory

Tertiary Hospital (non-hematological center)

Secondary Hospital

Other

(What is the duration of your overseas continuing education (for those who continuing educated overseas)) [Single-choice Questions] [Mandatory]

Less than 3 months

3 to 6 months

7 to 12 months

1 to 2 years

More than 2 years

(What is your main subspecialties? ) [Multiple-choice Question] [Mandatory]

Leukemia

Lymphoma

Plasmacyte disease

Erythrocytic disease

Thrombosis and haemostasis

Transplantation and cellular therapy

Inherited and genetic diseases

Other____

(Have you published any professional papers as the first or corresponding author in 2 years?) [Single-choice Questions] [Mandatory]

Yes

No

(Did you spend more time on research during the pandemic?) [Single-choice Questions] [Mandatory]

Yes

No

(What are your main sources of work pressure?) [Multiple-choice Question] [Mandatory]

Personal reasons

Family reasons

Hospital reasons

Others

(Do you have any intention to resign?) [Single-choice Questions] [Mandatory]

Yes

No

(If so, is your intention to resign related to the pandemic?) [Single-choice Questions] [Mandatory]

Yes

No

(If you were to resign, what would be your preferred choices? [Multiple-choice Question] [Mandatory])

Continuing education

Other public hospitals

Private hospitals

Research institutions

Medical-related enterprises

Non-medical industry

Other____

(Do you have any multisited practice or starting a secondary job?) [Single-choice Questions] [Mandatory]

Yes

No

Part Two: Diagnostic Data [Matrix Single-choice Questions] [Mandatory]

(The comparison of diagnostic data is between the pandemic period (January-April 2020) and the same period last year (January-April 2019).)

|  | *0-30%* | *30%-60%* | *>60%* | *No decrease* |
| --- | --- | --- | --- | --- |
| What is the percentage of patient visits you feel has decreased? | 🌕 | 🌕 | 🌕 | 🌕 |
| What is the percentage of out-of-town patient visits you feel has decreased? | 🌕 | 🌕 | 🌕 | 🌕 |
| What is the percentage of inpatients you feel has decreased due to the pandemic? | 🌕 | 🌕 | 🌕 | 🌕 |
| What is the percentage of available beds you feel has decreased due to the pandemic? | 🌕 | 🌕 | 🌕 | 🌕 |
| What is the percentage of patients whose treatment plans you feel has been changed due to the pandemic? | 🌕 | 🌕 | 🌕 | 🌕 |
| What is the percentage of hematological tumor patients whose chemotherapy plans you feel has been postponed due to the pandemic? | 🌕 | 🌕 | 🌕 | 🌕 |

During the pandemic (January-April 2020) compared to the same period last year, has the use of bone marrow stem cells for allogeneic transplantation decreased? [Single-choice Question] [Mandatory]

Decreased by 0-10%

Decreased by 11%-20%

Decreased by 21%-30%

Decreased by >30%

No decrease

During the pandemic (January-April 2020) compared to the same period last year, has the use of cord blood for allogeneic transplantation increased? [Single-choice Question] [Mandatory]

Increased by 0-10%

Increased by 11%-20%

Increased by 21%-30%

Increased by >30%

No increase

During the pandemic (January-April 2020) compared to the same period last year, what is the percentage of delayed autologous SCT? [Single-choice Questions] [Mandatory]

<10%

10-25%

＞25%

No delay

What are the main reasons for delaying allogeneic SCT? [Multiple-choice Question] [Mandatory]

Patient's condition

Hospital policy restrictions

Donor reasons

Patient's subjective willingness

Bed limitations

Other____
